# Gene-Activated Materials in Regenerative Dentistry: Narrative Review of Technology and Study Results

**DOI:** 10.3390/ijms242216250

**Published:** 2023-11-13

**Authors:** Olga Krasilnikova, Anna Yakimova, Sergey Ivanov, Dmitri Atiakshin, Andrey A. Kostin, Dmitry Sosin, Peter Shegay, Andrey D. Kaprin, Ilya Klabukov

**Affiliations:** 1 National Medical Research Radiological Centre of the Ministry of Health of the Russian Federation, Koroleva St. 4, 249036 Obninsk, Russia; 2 A. Tsyb Medical Radiological Research Centre—Branch of the National Medical Research Radiological Centre of the Ministry of Health of the Russian Federation, Zhukov St. 10, 249031 Obninsk, Russia; 3 Department of Urology and Operative Nephrology, Patrice Lumumba Peoples’ Friendship University of Russia (RUDN University), Miklukho-Maklay St. 6, 117198 Moscow, Russia; 4 Scientific and Educational Resource Center for Innovative Technologies of Immunophenotyping, Digital Spatial Profiling and Ultrastructural Analysis, Patrice Lumumba Peoples’ Friendship University of Russia (RUDN University), 117198 Moscow, Russia; 5 Centre for Strategic Planning and Management of Biomedical Health Risks of the Federal Medical Biological Agency, 119121 Moscow, Russia; 6 Obninsk Institute for Nuclear Power Engineering, National Research Nuclear University MEPhI, Studgorodok 1, 249039 Obninsk, Russia

**Keywords:** dentistry, gene activation, gene therapy, periodontitis, regenerative medicine, tissue engineering

## Abstract

Treatment of a wide variety of defects in the oral and maxillofacial regions requires the use of innovative approaches to achieve best outcomes. One of the promising directions is the use of gene-activated materials (GAMs) that represent a combination of tissue engineering and gene therapy. This approach implies that biocompatible materials will be enriched with gene-carrying vectors and implanted into the defect site resulting in transfection of the recipient’s cells and secretion of encoded therapeutic protein in situ. GAMs may be presented in various designs depending on the type of material, encoded protein, vector, and way of connecting the vector and the material. Thus, it is possible to choose the most suitable GAM design for the treatment of a particular pathology. The use of plasmids for delivery of therapeutic genes is of particular interest. In the present review, we aimed to delineate the principle of work and various designs of plasmid-based GAMs and to highlight results of experimental and clinical studies devoted to the treatment of periodontitis, jaw bone defects, teeth avulsion, and other pathologies in the oral and maxillofacial regions.

## 1. Introduction

Application of regenerative medicine and tissue engineering methods has great potential for improvement of oral and dental tissue regeneration and can be called regenerative dentistry [1]. Regenerative techniques have been studied for the treatment of periodontal disease, jaw bone and gingiva defects, age-related disorders, etc., with the aim of restoring tissue properties, functions, and structure. Since tissue regeneration in the oral and maxillofacial regions involves multifaceted and complex processes, the enhancement of tissue-engineering tools with gene therapy promises to improve outcomes. The concept of gene-activated materials (GAMs) represents the combination of tissue engineering and gene therapy [2]. The GAM approach implies that biocompatible materials will be enriched with vectors carrying therapeutic genes and will result in transfection of recipient’s cells after implantation [3,4].

Previous studies have shown that the use of various growth factors can increase clinical attachment level, reduce gingival recession, and enhance linear bone gain in patients with periodontal osseous defects, and stimulate proliferation of gingival fibroblasts, osteoblasts, periodontal ligament cells, etc. [5,6,7,8]. However, the benefits of growth factors face a problem of rapid protein degradation requiring the use of supraphysiological doses [9]. Transfection of cells with genes encoding therapeutic proteins helps to overcome these disadvantages and facilitates gradual prolonged protein secretion by recipients’ cells in the defect site converting them into bioreactors in situ [10].

Composition of plasmid-based GAMs implies the presence of the following essential elements: naked plasmid or plasmid/delivery system complex, encoded therapeutic protein, and biocompatible material, enriched with plasmid. The way of attaching plasmid to the material is also of importance since it may influence kinetics of plasmid release. Variability inside GAM elements allows achievement of different effects. Thus, various encoded proteins exert different therapeutic functions. Materials used for GAM creation may also possess different properties depending on chemical structure and biomechanical parameters that may influence tissue regeneration and clinical outcome [11,12,13]. The exact GAM composition may be chosen depending on the clinical task to be solved.

The use of GAM has already shown its potential in pre-clinical studies for the treatment of bone and skin defects [14,15]. In the field of regenerative dentistry, GAMs were successfully used in vivo for teeth replantation, treatment of alveolar bone defects after tooth extraction, and even showed their safety and efficacy for jaw bone grafting in clinical trial [16,17,18]. Osseointegration of titanium implants with the use of the GAM approach was also investigated in vivo [19].

There are various approaches to deliver genetic material including viral and non-viral delivery vectors. It is generally considered that viral vectors have higher transduction efficiency compared to plasmid vectors; however, viral vectors possess several disadvantages, such as immune response. At the same time, plasmids have lower immunogenicity, and their transfection efficiency may be enhanced using special delivery systems [20]. Considering the advantages of non-viral delivery, in the present review, we will discuss GAMs created with the use of naked plasmids and non-viral delivery systems. GAMs are frequently referred to as gene-activated matrices; however, since genetic activation of titanium implants is also described in the present review, we will use a wider term ‘gene-activated materials’. This work represents a narrative review for which we searched the PubMed database to find studies on the GAM approach in regenerative dentistry. In the present narrative review, we described results of clinical, in vivo, and in vitro studies in which plasmid or a plasmid/delivery system complex was used in combination with materials in dentistry-related studies. We did not analyze results of the studies that used viral vectors, plasmid vectors without materials, and articles in which plasmid was used to directly transfect cells under in vitro conditions.

Despite a relatively small number of published works on GAMs in the field of regenerative dentistry, there is a substantial heterogeneity in GAM design. Various materials, encoded proteins, and delivery systems, as well as methods of attaching plasmid to the material, were used for GAM creation. In the present review, we will discuss works on clinical and pre-clinical use of plasmid-based GAMs in regenerative dentistry and analyze various approaches to their creation.

## 2. Gene-Activated Materials’ Mechanism of Action

GAMs’ mechanism of action is based on the combination of the properties of the material to form a provisional template for new tissue formation in the area of the defect and the properties of a gene-carrying vector to transfect recipient’s cells and stimulate local secretion of encoded protein. Exact mechanisms of action are deeply dependent on pathology being addressed, type of biocompatible material used, encoded protein, type of delivery system, and approach to attaching plasmid (or a plasmid/delivery system complex) to the material.

**Figure 1:**
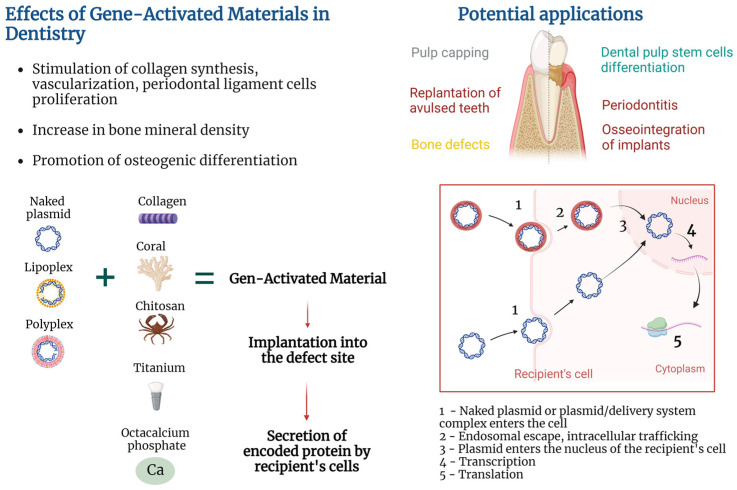
Gene-activated materials’ mechanism of action. Created with BioRender.com.

Transfection of cells may be performed in vitro by culturing cells with non-viral vectors, followed by seeding of cells on the material and implantation into the damaged area [21]. However, the GAM approach implies that the material will be enriched with plasmid or a plasmid/delivery system complex (in contrast to preliminary transfected cells), implanted into the damaged area, followed by the recipient’s cell transfection and secretion of encoded protein (Figure 1). Such an approach helps to elude the costly and labor-intensive stage of in vitro cell culture and does not fall under strict legal requirements for cell culture.

Every particular event that occurs upon GAM implantation represents a separate and complex field of extensive research. Next, events that occur upon GAM implantation will be briefly delineated.

### 2.1. Implantation of GAM into a Damaged Area

The technique of GAM positioning at the injury site depends on material and treated pathology. For example, fixation of gene-activated octacalcium phosphate (OCP) within a bone grafting site was achieved via soft tissue suturing in a clinical trial [18]. In vivo studies also demonstrated various techniques of GAM implantation; for example, covering of the root of avulsed teeth with poly(D,L-lactic-co-glycolic acid) (PLGA) in dogs [16], placing collagen into an alveolar bone defect followed by muscle suturing and skin incision closure with clips in rats [22], filling of sockets after tooth extraction with gelatin sponges followed by coverage with periodontal dressing in rats [17], and layer-by-layer coating of a titanium implant followed by implantation in dogs [19].

### 2.2. Colonization of the Material with Cells/Release of the Plasmid from the Material

After implantation, the GAM starts to exert dual functions explained by a combination of tissue engineering and gene therapy in the GAM approach. Aside from being tools for incorporation of the plasmid or plasmid/delivery system complex, the paradigm of tissue engineering implies that materials should represent a provisional template for cell migration, adhesion, proliferation, differentiation, and deposition of extracellular matrix at the defect site [2]. Release of the plasmid or plasmid/delivery system complex from GAM occurs in the course of material degradation or plasmid diffusion depending on the type of material and method of plasmid attachment [4]. Tailoring physicochemical parameters of the materials, as well as choosing ways of adhering plasmid to the material, allow the achievement of different plasmid release kinetics, which is valuable for temporal control over gene expression [23,24].

### 2.3. Recipient’s Cell Transfection

Transfection of cells occurs via uptake of naked plasmids of the plasmid/delivery system complex. Some works used naked plasmids for cell transfection in vivo and in vitro, suggesting their ability to be internalized and result in increased protein secretion [25], or in reporter gene expression [4]. At the same time, there is an opinion that entry of naked plasmids into eukaryotic cells may be hampered by the negative charge of the plasmid, so a variety of approaches were used to increase transfection efficiency [26]. For example, cationic polymers facilitate plasmid/delivery system complex uptake via neutralization of DNA negative charge followed by endocytosis and endosomal escape [27].

### 2.4. Cytoplasmic Transport and Nucleus Entry

After entering the cell, free DNA is complexed with intracellular proteins that facilitate microtubule-mediated cytoplasmic transport of plasmid DNA to the surface of the cell nucleus [28,29]. Plasmids may enter the nucleus of dividing cells at the time of nuclear envelope disassembly or cross the nuclear envelope of non-dividing cells via nuclear pore complex [28,30].

### 2.5. Transcription, Translation, Expression Duration

The host’s RNA polymerase II facilitates plasmid DNA transcription and mRNA synthesis. The duration of transgene expression greatly depends on the cell type that was transfected. For example, muscle cells are characterized by long-lasting expression in mice for more than a year, presumably because of the absence of cell division [31]. When GAMs are concerned, duration of expression also depends on plasmid release kinetics from the materials, with a longer release period yielding prolonged expression [24].

### 2.6. Protein Secretion

Transfected cells start to secrete plasmid-encoded proteins which may be directed to the achievement of various goals. For example, chitosan/collagen matrix enriched with plasmid encoding platelet-derived growth factor (pPDGF) promoted proliferation of human periodontal ligament cells (hPDLCs) and formation of a periodontal connective tissue-like structure in vitro [4]. At the same time, Yang et al. reported slower proliferation of dental pulp stem cells (DPSCs) cultured on chitosan/collagen matrix enriched with plasmids carrying the bone morphogenetic protein-7 (BMP) gene and hypothesized that this may be due to the reverse correlation between the DPSCs’ odontoblastic differentiation caused by BMP-7 secretion and proliferation [32].

## 3. Heterogeneous Design of Gene-Activated Materials

In the field of regenerative dentistry, there is heterogeneity in the design of plasmid-based GAMs. It is explained by the diversity in the following fundamental structural elements of the GAMs: (1) type of material used, (2) type of encoded protein, (3) type of delivery system, (4) method of attaching plasmid or plasmid/delivery system complex to the material. The exact GAM design may be chosen considering the type of treated pathology and individual patient characteristics. Next, we will briefly characterize structural elements that were used for GAM creation in the field of regenerative dentistry.

### 3.1. Materials Used for Creation of GAMs in the Field of Regenerative Dentistry

In tissue engineering, a variety of natural and synthetic materials are used. In order to be used for creation of a GAM, material must not only be biocompatible and support tissue regeneration but also be able to attach a naked plasmid or a plasmid/delivery system complex. To create GAMs in the field of regenerative dentistry, various materials are used depending on the scientific and clinical problem being solved.

Collagen is a biocompatible biodegradable material capable of stimulating migration of human oral region fibroblasts, endothelial, and periodontal ligament cells [33], and healing of bone, cementum, and periodontal ligament [34], and alveolar bone defects [35]. Chitosan is a natural biocompatible, biodegradable polymer with a low immunogenicity possessing bacteriostatic, fungistatic, and anti-inflammatory properties [36,37], capable of promoting the proliferation of human hPDLCs [38].

Hydroxyapatite is frequently used for bone grafting due to the resemblance of its structure and composition to the natural mineral phase of human bone tissue, and osteoconductive and osteoinductive properties [39]. In dentistry, hydroxyapatite is frequently combined with collagen to achieve enhanced mechanical properties [40].

Coral is a natural porous biocompatible material also used for bone tissue engineering due to its osteoconductive and mechanical properties [41]. Coral scaffolds gene-activated with pPDGF-B were shown to stimulate proliferation of hPDLCs and synthesis of type I collagen, suggesting its possible use for periodontal regeneration [42].

Poly(lactic-co-glycolic acid) is a biocompatible and biodegradable copolymer of lactic and glycolic acid. Depending on the composition and manufacturing conditions, PLGA may be tailored to have a certain degradation and drug/plasmid release rates [43,44], mechanical properties, and porosity [45].

Gelatin is a biocompatible biodegradable natural bio polymer characterized by cost-effectiveness and low immunogenicity, as well as various physical and chemical properties depending on the preparation technique [46].

Octacalcium phosphate (OCP) is a mineral precursor of apatite crystals in bone tissue; it possesses osteoconductive and osteoinductive properties and allows adhesion of bioactive molecules [47].

Titanium and its alloys are widely used in dental prosthetics and for the reconstruction of maxillofacial bone defects as well as in orthopedics [48,49]. Despite excellent biocompatibility, surface modifications of titanium implants are studied to improve their osseointegration [48].

### 3.2. Types of Encoded Proteins Used in GAMs in Regenerative Dentistry

The importance of growth factors in soft and bone tissue regeneration has already been shown in multiple studies. Next, various proteins encoded by plasmids in GAMs used in regenerative dentistry will be briefly overviewed.

Vascular endothelial growth factor (VEGF) not only acts as a mitogen for endothelial cells stimulating angiogenesis but also promotes human periodontal ligament stem cells’ (hPDLSCs) odonto-/osteogenic differentiation in vitro [50,51]. Genetic activation of materials with pVEGF is a promising direction for the stimulation of bone tissue regeneration since sufficient vascularization is necessary for successful osteogenesis [18].

Platelet-derived growth factor is capable of stimulating osteoblast, periodontal ligament cell, and gingival fibroblast proliferation [6,8]. Clinical trials showed that PDGF in combination with beta-tricalcium phosphate resulted in an increased clinical attachment level and reduced gingival recession at 3 months, and greater linear bone gain and defect fill at 6 months compared to scaffold alone [7].

Fibroblast growth factors (FGFs) are a large family of regulatory molecules that have promoting effects on a variety of cells including hPDLCs and hPDLSCs [5,51]. An in vitro study with the use of a mouse periodontal ligament cell line also showed the ability of FGF-2 to stimulate VEGF-A secretion [52]. Notably, recombinant human FGF-2 in combination with modified Widman periodontal surgery increased bone fill in patients with periodontitis in a clinical trial [53].

Bone morphogenetic proteins are signaling molecules with multiple functions and members of the TGF-β superfamily [54]. BMP-2 is used for the treatment of bone lesions since this protein possesses high osteogenic activity via promoting osteogenic differentiation of pre-osteoblastic cells [55,56].

Although the benefits of using growth factors have been shown in many studies, their use has some disadvantages, including a short half-life when applied to the defect site and application of supraphysiological doses [9]. The use of GAMs allows for the prevention of the problem of rapid degradation of recombinant growth factors as well as for gradual secretion of the growth factor by the recipient’s cells at the defect site.

### 3.3. Types of Non-Viral Delivery Systems Used in GAMs in the Field of Regenerative Dentistry

Ensuring the efficient transfer of genetic material into the cell is one of the main challenges in gene therapy. To enhance plasmid transfection efficiency, a variety of non-viral delivery agents are used. Non-viral delivery systems may be represented by biodegradable and non-biodegradable polymers, lipids, peptides, inorganic materials (e.g., gold and magnetic nanoparticles), hybrid systems [57], calcium phosphate [58], exosomes [59], etc. We will briefly describe non-viral delivery systems that are used as part of GAMs in the field of regenerative dentistry.

Polyethyleneimine (PEI) is a positively charged cationic polymer that facilitates plasmid entry through negatively charged cell membranes via condensation of negatively charged plasmid DNA [60]. One of the most common cationic polymers is represented by PEI, with 25 kDa branched PEI being considered one of the most efficient types [17]. However, the non-biodegradable nature of PEI may result in a cytotoxic effect as seen in human periodontal ligament fibroblasts and gingival fibroblasts [22]. Some authors suggested that cytotoxicity is higher in PEI with high molecular weight compared to low molecular weight [61]. Importantly, Plonka et al. showed that even though PEI reduced viability of human periodontal ligament fibroblasts and gingival fibroblasts, transfection of PEI/pPDGF polyplexes nivelated detrimental impact of PEI suggesting the stimulating effect of transfection with growth factor-encoding plasmid [22].

Chitosan is a natural cationic biodegradable polymer capable of interacting with negatively charged plasmid DNA via electrostatic forces [62]. Low cytotoxicity was also reported for the chitosan-based delivery system [63]. In the study by Peng et al. (2009), transfection of hPDLCs with pDNA/chitosan resulted in higher cell viability compared to naked plasmid alone [4]. At the same time, many studies combined chitosan with other delivery systems or subjected it to various modifications to improve its transfection efficiency [64].

Cationic liposomes are widely used gene delivery systems. Cationic liposomes act by binding positively charged head groups of lipids with negatively charged phosphate groups of plasmid DNA and by surrounding DNA molecules [65].

Delivery of naked plasmid DNA is also considered as a promising direction and has some advantages, among which is the absence of delivery systems with potential cytotoxicity. Supercoiling of plasmid DNA also aids more efficient transfection [66].

### 3.4. Methods of Binding Plasmids or Plasmid/Delivery System Complexes to the Materials

Various methods of attaching the plasmid or plasmid/delivery system complex to the material are used and are of importance since this parameter may affect the kinetics of plasmid release and duration of transgene expression [24]. Methods of attaching plasmids or plasmid/delivery system complexes to materials may be divided into chemical and physical methods. Chemical methods rely on the creation of bonds that facilitate plasmid attachment to the material. For example, calcium-containing materials may bind DNA via electrostatic interactions due to the presence of phosphate groups on the DNA backbone.

Physical methods do not imply creation of bonds between plasmid and material and include emulsion electrospinning that allows for the incorporation of the buffer with the target plasmid into the volume of polymeric microfibers for subsequent release in the course of material degradation [16,67]. Importantly, several authors noted that incorporation of the plasmid into the material may cause DNA damage due to interaction with solvents during manufacture [2,24]. One of the solutions to this issue is coaxial (core-shell) electrospinning that allows for the obtaining of core-shell fibers, the inner compartment of which may be filled with a plasmid-containing buffer avoiding plasmid interaction with damaging solvents [24].

From a technical point of view, the attachment of a naked plasmid or a plasmid/delivery system complex to the material may be achieved by different methods, some of which were described by Peng et al. [4]. Among such methods are dropping plasmid-containing suspension on the material or soaking of the material in the suspension followed by some time of incubation [17,68], injection into the volume of the material [69], or coating of the material with other plasmid-containing materials [42]. The layer-by-layer technique allows for the coating of the materials with multiple oppositely charged plasmid-containing layers [19].

## 4. Plasmid-Based Gene-Activated Materials in Regenerative Dentistry: Preclinical and Clinical Results

GAMs of various designs were investigated in vitro, in vivo, and in clinical studies for potential treatment of periodontitis, jaw bone defects, dental pulp capping, gene-activation of titanium implants, improvement of teeth replantation, etc. Despite the growing interest towards gene therapy and the use of GAMs in different fields, the overall number of studies on GAMs in the field of regenerative dentistry is not large (Table 1).

### 4.1. Periodontitis

Periodontitis is a multifactorial inflammatory disease of the oral cavity that remains one of the greatest challenges in modern dentistry [77]. This condition is characterized by inflammation and progressive destruction of supportive tissues of teeth including gingival tissues, periodontal ligament, and in severe cases—of alveolar bone which can result in tooth loss [78]. Bacterial colonization is considered to be one of factors triggering immune response and development of periodontitis [79].

Human periodontal ligament cells are mainly presented by fibroblasts and are one of the key players needed for periodontitis treatment since they surround the teeth and attach them to the inner wall of alveolar bone. Impacts of GAMs with various designs on hPDLCs were studied in various in vitro and in vivo studies. An increase in collagen synthesis represents a positive sign since collagen is one of the main proteins of periodontal ligament attaching the tooth to the alveolar bone [80]. In one of the early GAM studies, Zhang et al. [42] demonstrated that a coral composite scaffold gene-activated with the mixture of plasmid encoding platelet-derived growth factor beta (pPDGF-B) and chitosan solution increased hPDLC proliferation and levels of PDGF-B and collagen mRNA expression in vitro.

However, it was previously shown that chitosan/collagen scaffolds enriched with a viral vector carrying the *TGF-β1* gene resulted in greater secretion of TGF-β1, and higher levels of hPDLC proliferation and collagen type I and III mRNA compared to the plasmid-enriched scaffolds [68]. Higher transduction efficiency of viral vectors compared to naked plasmid is a known occurrence; at the same time, plasmids possess other advantages compared to viral vectors which make plasmids valuable tools for potential clinical applications. Moreover, various plasmid delivery systems are capable of enhancing transfection efficiency. At the same time, cell viability is also one of major concerns when delivery vectors are used. Peng et al. reported that hPDLCs cultured on a chitosan/collagen scaffold enriched with chitosan/pPDGF nanoparticles had better viability and demonstrated increased scaffold pore colonization compared to scaffold enriched with naked plasmid without a chitosan delivery system [4]. Importantly, cell-seeded scaffolds enriched with chitosan/pPDGF nanoparticles displayed formation of a periodontal-like structure after 2 weeks of in vitro culture [4]. These results suggest the valuable role of chitosan delivery systems in facilitation of plasmid protection from degradation and cell entry.

Advanced and severe stages of periodontitis are also characterized by alveolar bone destruction, which is one of the main challenges in dentistry [81]. The use of GAMs for the regeneration of destructed bone tissue may represent a valuable approach since it is possible to use various osteoconductive materials enriched with genes encoding proteins with shown osteoinductive properties. Achieving prolonged gene expression and osteoinductive protein secretion in the defined region may contribute to bone healing. In the study by Xie et al., hPDLSCs were cultured on a PLGA coaxial scaffold with a core layer of plasmid carrying *BMP-2* gene (pBMP-2) and PEI and on single axial electrospun PLGA scaffold with the same plasmid/delivery system complex embedded [24]. Study results showed prolonged expression of BMP-2 in coaxial scaffolds [24].

One of the important tasks in gene therapy is the determination of the optimal time during which the synthesis of the target protein will yield best results. Reparative processes in the body are finely orchestrated and the synthesis of a particular protein can have a positive effect on tissue regeneration if it occurs during a physiologically appropriate time [82,83]. In the study by Plonka et al. on the treatment of periodontal alveolar defects in rats, it was shown that bridging bone length and new bone area were significantly smaller in the group receiving collagen gene-activated with PEI/pPDGF-B compared to groups treated with collagen or recombinant human PDGF-BB alone [22]. The authors concluded that prolonged secretion of PDGF-BB may be associated with inflammation and that a short-term secretion could be more beneficial [22]. Thus, there is a need to further investigate possible reasons for the absence of positive results and determine time intervals during which the secretion of particular therapeutic protein will be physiologically useful.

### 4.2. Teeth Replantation

The need for teeth replantation may arise in cases of traumatic tooth avulsion [84]. External root resorption of replanted teeth was documented to be a frequent complication of this procedure [16,85]. The formation of periodontal ligament around a replanted tooth is suggested to be one of important factors for achieving successful replantation and reducing the risk of root resorption [86]. Jiang et al. showed that covering roots of replanted teeth in beagles with PLGA enriched with PEI/pFGF-2 resulted in the reduced root resorption compared to PLGA alone [16]. In vitro experiments also showed higher expression levels of Collagen I and scleraxis in hPDLCs cultured with gene-activated PLGA compared to PLGA alone and no difference in alkaline phosphatase between two types of materials [16].

### 4.3. Jaw Bone Defects and Alveolar Ridge Atrophy

Bone defects may appear in the oral and maxillofacial regions due to various reasons, including trauma, tumors, cysts, apicectomies, severe periodontitis, etc. [87].

The first case of clinical use of GAM for the treatment of mandible bone defects was presented by Bozo et al. in 2016 [72]. Collagen-hydroxyapatite scaffold was gene-activated with plasmid DNA encoding VEGF-165 and used to treat a patient with non-unions of a previously reconstructed mandible [72]. The authors reported successful consolidation and bone formation in place of one non-union out of two and reported the absence of adverse events [72]. Bozo et al. also reported results of a clinical trial involving 20 patients with atrophy of alveolar ridges and jaw bone defects who received bone grafting with OCP bone substitute gene-activated with plasmid encoding VEGF-165 [18]. Trial results demonstrated formation of a new bone tissue (average bone density of 908.13 ± 114.40 HU) in all patients 6 months after implantation of gene-activated OCP. Importantly, the authors also reported the absence of adverse events during a follow-up period of 30 months [18].

Studies investigating effects of various plasmid doses are of particular importance. Thus, in the study by Kolk et al., titanium discs were gene-activated with various concentrations of plasmid in copolymer-protected PEI/pBMP-2 polyplexes and coated with poly(D,L-lactide), and used for the treatment of critical-size mandibular bone defects in a rat model [73]. Importantly, results of this study demonstrated inverse dose–response with maximal plasmid concentrations (50 and 100 µg) not leading to defect coverage; meanwhile, a concentration of 2.5 µg yielded complete defect closure at day 112. Treatment with GAM containing 2.5 µg of plasmid also resulted in higher mineral density compared to the group receiving GAMs with 100 µg of plasmid at day 112 [73]. The demonstrated results highlight the need for thorough investigation of the effects of different growth factors in different doses to achieve improved outcomes.

Works comparing effects of recombinant growth factors and plasmids are of utmost value and deserve the closest attention. In continuation of the previous work, Kolk et al. used poly(D,L-)lactide-coated titanium discs enriched with either copolymer-protected PEI/pBMP-2 or recombinant human BMP-2 [71]. Complete consolidation of critical-size mandibular bone defects in rats was achieved in both groups. The recombinant protein group was characterized by a higher bone volume at all time points; however, an assessment of the quality of a newly formed bone showed that mineral density was higher in the gene-activated group at later time points [71]. As can be seen from the study results, both approaches may be further improved with the goal of achieving faster but also more physiologically appropriate new bone formation in critical-size mandibular defects.

Application of novel methods in regenerative dentistry is of particular interest. For example, 3D printing shows great promise for the creation of personalized scaffolds that will precisely match the configuration of the defect [88,89]. In the study by Bozo et al., 3D printed OCP scaffold was gene-activated with plasmid DNA encoding VEGF-165 and implanted in the zone of mandible defects in pigs [70].

Tooth extraction may also lead to alveolar bone loss, conditioning the need for restoration of bone and soft tissues [90]. In the study on alveolar bone regeneration in a tooth extraction model in rats by Jin et al., absorbable gelatin sponge enriched with PEI-alginate/pBMP-2 was implanted into post-extraction sockets [17]. Study results displayed a higher residual alveolar ridge, more pronounced bone formation, and higher bone mineral density in the defect site compared to non-gene-activated gelatin sponge at weeks 4 and 8 [17].

### 4.4. Implantology

Dental implant placement has become a common practice with high success rates. At the same time, avoidance of peri-implant diseases and acceleration of osseointegration still remain clinical tasks of utmost importance [19].

Peri-implant diseases are associated with inflammation of the tissues around the implant and are typically caused by bacterial plaque [91]. Rapid recovery of the soft tissues around the implant is important, since it aids protection against bacterial colonization. In order to enhance the soft tissue seal around the titanium implant and to prevent bacterial load, titanium enriched with PEI/pPDGF-B was studied in vitro by Laird et al. [74]. Study results showed that gene-activated titanium led to the elevated PDGF-BB secretion in human primary gingival fibroblasts and increased levels of integrin-α2 mRNA expression—a gene related to the capacity of cells to adhere to titanium and to form a soft tissue seal [74].

Osseointegration of titanium implants is one of the prerequisites for successful dental implantation. The titanium surface undergoes various modifications to enhance osseointegration [92]. Coating with growth factors, including PDGF and BMP-2, was also investigated [93,94]. The coating of titanium implants with plasmids encoding BMP-2 represents an alternative to the use of recombinant proteins. Atluri et al. showed that titanium coated with PEI/pBMP-2 nanoplexes led to an increased BMP-2 and pro-osteogenic alkaline phosphatase expression in human bone marrow mesenchymal stem cells on day 7 in vitro compared to cells cultured with titanium alone and titanium enriched with pBMP-2 without a delivery system [75]. Increased mineralization as well as calcium ion deposition were also demonstrated, suggesting potential of this GAM approach for enhancement of implant osseointegration [75].

One of the promising directions in GAM creation is a layer-by-layer coating technique that allows the adherence of multiple layers of a plasmid or plasmid/delivery vector complex. Thus, Jiang et al. [76] showed that titanium implants coated with multiple layers of hyaluronic acid and liposome/pBMP-2 stimulated expression of alkaline phosphatase (ALP) and secretion of osteocalcin in pre-osteoblastic cells, suggesting their osteogenic differentiation and prospectiveness for use in implantology. However, further in vivo study with the use of titanium enriched with liposome/pBMP-2 by He et al. showed no statistically significant differences in bone-to-implant contact during 12 weeks and in intrathread bone area in comparison to non-gene-activated implants in dogs [19]. The closer investigation of possible reasons for the absence of improvements in vivo is needed, possibly with the use of other encoded proteins.

### 4.5. Tooth Pulp Capping and Impact on Dental Pulp Stem Cells

Dental pulp represents an unmineralized connective tissue comprising peripheral nerves, capillary blood vessels, lymphatic elements, and fibroblastic and stem cells [95]. Pulp capping systems are needed to protect the pulp from bacteria and maintain its vitality [96], and mineral trioxide aggregate (MTA) is often used for pulp capping purposes [97]. At the same time, the idea of creating a bioactive capping system that will be able to promote regeneration of damaged tooth tissues is attractive, especially considering the presence of dental pulp-derived stem cells. Chakka et al. showed that the pulp capping system from a collagen/pBMP-2/pFGF-2/PEI complex stimulated migration of human DPSCs towards a gene-activated tooth cap without signs of necrosis observed in the MTA group in a model of extracted human molars [69]. Important results regarding performance of different encoded genes and their combination were also obtained. pBMP-2/pFGF-2/PEI tooth caps resulted in lower expression levels of dentin matrix protein 1 compared to MTA, suggesting less effective promotion of differentiation [69]. At the same time, a PEI/pBMP-2-activated tooth cap resulted in levels of dentin sialophosphoprotein expression comparable with MTA [69]. The obtained results highlight the need for a thorough study and selection of encoded proteins and their combinations.

Stem/stromal cells were successfully isolated from the gingiva, periodontal ligament, dental pulp of permanent and deciduous teeth, buccal fat pad, dental follicle, and apical papilla of developing teeth [98]. The use of stem cells was actively studied in the field of regenerative dentistry due to their multilineage differentiation potential and immunomodulatory functions. Previous works showed the ability of DPSCs to differentiate into odontoblasts [99,100] and form osteodentin-like structures during heterotopic implantation in vivo [101]. However, we have not found any work showing the ability of native DPSCs to repair damaged dentin in the same tooth in situ.

The use of GAMs was also studied in order to assess their impact on DPSC behavior. Chitosan/collagen scaffold enriched with pBMP-7 was shown to stimulate human DPSCs as was seen in the higher values of ALP activity and calcium content in comparison with non-activated materials [32]. Of note, DPSCs cultured on GAM showed slower proliferation compared to those cultured on chitosan/collagen alone, possibly due to the inverse relationship between proliferation and differentiation. Although the stimulating effect of GAM on DPSC differentiation was shown in vitro, there is a need to conduct further animal studies in order to choose optimal conditions for the activation of residing DPSC differentiation potential and achievement of regeneration of damaged tooth tissue in situ.

## 5. Limitations of Plasmid-Based GAM Approach

In vitro studies have shown positive effects of GAMs on behavior of various cells, and animal studies also demonstrated positive results of GAM use for experimental treatment of certain conditions. For example, GAM application led to the reduced root resorption of replanted teeth in dogs and enhanced alveolar bone regeneration after tooth extraction in rats [16,17]. At the same time, in the model of implantation in dogs, genetic activation of titanium with liposome/pBMP-2 did not significantly improve bone-to-implant contact and intrathread bone area compared to the control group [19]. Such heterogeneous results suggest the need for further studies dedicated to the treatment of specific pathologies in order to establish the most beneficial GAM design that will maximally contribute to the treatment of specific pathology.

GAMs consist of various elements and may include not only naked plasmids but also plasmids with special delivery systems intended to enhance transfection efficiency. At the same time, the use of particular types of delivery systems may be associated with cytotoxic effects and immune response from the recipient [65,102,103,104]. Cell-based approaches were also studied in various fields including dentistry, chronic wound treatment, etc. [105,106]. Notably, GAMs allow for the avoidance of possible adverse events and side effects related to the cell therapy [107,108].

## 6. Future Directions

Since regeneration of various tissues is accompanied by finely orchestrated expression of particular genes at certain periods of a regenerative process, it can be concluded that there is a need to clearly select the time points and intervals during which gene expression and secretion of encoded therapeutic proteins will be physiologically useful. As evidenced in the study by Plonka et al., the use of GAM encoding PDGF-B did not result in accelerated regeneration of periodontal alveolar tissues in rats [22], which may be related to the unnecessary prolonged secretion of this growth factor.

One of the most valuable properties of GAMs is a provision of spatiotemporal control over plasmid release via choosing appropriate methods of plasmid attachment to the material and parameters of material degradation [23]. The promising direction in the field of GAMs is creation of multilayered materials [109] or multilayered coatings with different layers containing plasmids that encode different genes [110]. This approach may enable the sequential release of various plasmids at specific time points during the regenerative process, facilitating the synthesis of particular proteins needed at certain stages of regeneration. However, further studies are required to develop novel materials that will facilitate reliable and finely controlled release of plasmids according to the stage of the regenerative process. At the same time, the question of the stability of the plasmid inside the material during a prolonged period of time requires further investigation [23].

Among studied advanced approaches for gingival tissue regeneration is the use of collagen materials [111]. At the same time, we have not found papers on the treatment of gingival defects with the use of gene-activated collagen even though work showing that collagen may be enriched with plasmid DNA was published [112]. Considering the wide application of collagen in dental practice, its enrichment with gene constructs may yield promising results, but further thorough investigation of such a potential approach is still needed. The GAM approach is also valuable for development of in situ tissue engineering considering that genetic activation of materials may be performed in the operating room [113].

A promising direction that deserves further investigation is the creation and the use of delivery systems allowing cell-specific transfection of particular cell types in the oral and maxillofacial regions. This approach may find application during avulsed teeth replantation when stimulation of periodontal ligament regeneration may be achieved with selective transfection of periodontal ligament cells without stimulating osteoblasts for prevention of ankylosis. Selective cell transfection may possibly be achieved with the use of antibody-containing delivery systems [114]; however, we did not find works on creation of GAMs in the field of regenerative dentistry with the use of antibody technology. Investigation of a wider range of encoded proteins (besides growth factors) may also be useful. For example, poor bone healing may be associated with reduced expression of cyclooxygenase 2 warranting future research [115].

Further research is needed to investigate GAMs’ potential to a full extent and to establish the most beneficial GAM designs for regeneration of specific tissues.

## 7. Conclusions

GAMs are composed of multiple elements and exhibit substantial variability in the design. To achieve the best results, it is critical to carefully select an optimal combination of materials, encoded proteins, plasmid concentrations, and delivery systems in the composition of a GAM directed to regeneration of specific tissue.

Plasmid-based GAMs represent valuable tools in regenerative dentistry, allowing for the gradual prolonged secretion of therapeutic proteins. Genetic activation of materials with plasmids is of great interest since plasmids allow for the avoidance of disadvantages of viral vectors. Development of novel materials, delivery systems, plasmids, and methods of attaching genetic constructs to the materials, as well as conduction of new comparative studies, will facilitate further progress.

## Figures and Tables

**Table 1:** Studies with the use of plasmid gene-activated materials in the field of regenerative dentistry.

	Author	Encoded Protein	Delivery System/Vector	Material	Study Design
Periodontitis					
1	Xie et al. (2016) [24]	BMP-2	PEI/plasmid	PLGA	in vitro in human periodontal ligament stem cells
2	Plonka et al. (2016) [22]	PDGF-B	PEI/plasmid	collagen	in vitro in human periodontal ligament and gingival fibroblasts, in vivo in rats
3	Peng et al. (2009) [4]	PDGF-B	chitosan/plasmid	chitosan/collagen	in vitro in human periodontal ligament cells
4	Zhang et al. (2007) [42]	PDGF-B	chitosan/plasmid	coral	in vitro in human periodontal ligament cells, in vivo in mice
5	Zhang et al. (2006) [68]	TGF-β1	-/plasmid -/viral	chitosan/collagen	in vitro in human periodontal ligament cells, in vivo in mice
Teeth replantation					
6	Jiang et al. (2020) [16]	FGF-2	PEI/plasmid	PLGA	in vitro in human periodontal ligament cells, in vivo in dogs
Jaw bone defects and alveolar ridge atrophy					
7	Bozo et al. (2021) [18]	VEGF-165	-/plasmid	octacalcium phosphate	in vitro in BM-MSCs, in vivo in mice and rabbits, open-label non-randomized clinical trial
8	Bozo et al. (2020) [70]	VEGF-165	-/plasmid	3D printed octacalcium phosphate	in vivo in rats, mice, and pigs
9	Kolk et al. (2019) [71]	BMP-2	PEI/plasmid	PDLLA-coated titanium	in vivo in rats
10	Jin et al. (2019) [17]	BMP- 2	PEI-alginate/plasmid	gelatin	in vitro in MC3T3-E1 pre-osteoblasts, in vivo in rats
11	Bozo et al. (2016) [72]	VEGF-165	-/plasmid	collagen-hydroxyapatite	clinical case
12	Kolk et al. (2016) [73]	BMP-2	PEI/plasmid	PDLLA-titanium	in vivo in rats
Implantology					
13	Laird et al. (2020) [74]	PDGF-B	PEI/plasmid	titanium	in vitro in HEK293T cells and human primary gingival fibroblasts
14	Atluri et al. (2017) [75]	BMP-2	PEI/plasmid -/plasmid	titanium	in vitro in BM-MSCs
15	He et al. (2013) [19]	BMP-2	cationic liposome/plasmid	titanium	in vivo in dogs
16	Jiang et al. (2012) [76]	BMP-2	cationic liposome/plasmid	titanium	in vitro in pre-osteoblastic MC3T3-E1 cells
Tooth pulp capping and impact on dental pulp stem cells					
17	Chakka et al. (2020) [69]	BMP-2, FGF-2	PEI/plasmid	collagen	in vitro in dental pulp stem cells and in MC3T3 cells, ex vivo in extracted third molars
18	Yang et al. (2011) [32]	BMP-7	-/plasmid	chitosan/collagen	in vitro in dental pulp stem cells, in vivo in mice

BM-MSC, bone marrow mesenchymal stromal cell; BMP-2, bone morphogenetic protein 2; BMP-7, bone morphogenetic protein 7; FGF-2, fibroblast growth factor 2; PDGF-B, platelet-derived growth factor B; PEI, polyethyleneimine; PDLLA, poly(D,L-lactic acid); PLGA, poly(D,L-lactic-co-glycolic acid); TGF-β1, transforming growth factor beta 1; VEGF-165, vascular endothelial growth factor 165.

## Data Availability

Not applicable.
